# Functional Assessment of Chronic Illness Therapy-Fatigue is a reliable and valid measure in patients with active ankylosing spondylitis

**DOI:** 10.1186/s41687-022-00508-0

**Published:** 2022-09-23

**Authors:** David Cella, William R. Lenderking, Peter Chongpinitchai, Andrew G. Bushmakin, Oluwaseyi Dina, Lisy Wang, Joseph C. Cappelleri, Victoria Navarro-Compán

**Affiliations:** 1 Department of Medical Social Sciences, Northwestern University Feinberg School of Medicine, 625 North Michigan Avenue, Suite 2100, Chicago, IL 60611 USA; 2 Evidera, Waltham, MA USA; 3 Evidera, Bethesda, MD USA; 4 Pfizer Inc, Groton, CT USA; 5 Pfizer Inc, New York, NY USA; 6 IdiPAZ, University Hospital La Paz, Madrid, Spain

**Keywords:** Arthritis, Patient-reported outcome measures, Spondylitis, Ankylosing

## Abstract

**Background:**

The Functional Assessment of Chronic Illness Therapy-Fatigue (FACIT-F) scale has demonstrated good internal consistency and responsiveness to changes in clinical status among patients with ankylosing spondylitis (AS). We aimed to further evaluate the psychometric properties of the FACIT-F scale in adult patients with AS.

**Methods:**

Measurement properties of the FACIT-F scale were evaluated using data from tofacitinib phase 2/3 (NCT01786668/NCT03502616) studies in adult patients with active AS.

**Results:**

Second-order confirmatory factor modeling supported the measurement structure of the FACIT-F scale (Bentler’s comparative fit index ≥ 0.91), and FACIT-F demonstrated excellent internal consistency (Cronbach’s coefficient α ≥ 0.88) and test–retest reliability (Intraclass Correlation Coefficient ≥ 0.75). Correlation coefficients between FACIT-F and other patient-reported outcomes generally exceeded 0.40, supporting convergent validity. Meaningful within-patient change was estimated as 3.1–6.3 for FACIT-F total score, and 1.4–2.8 and 1.7–3.6 for FACIT-F Experience and Impact domain scores, respectively. Large (effect size ≥ 1.17 standard deviation units), statistically significant differences in FACIT-F domain/total scores between ‘no disease activity’ (Patient Global Assessment of Disease Activity [PtGA] = 0) and ‘very active disease’ (PtGA = 10) patient groups supported known-groups validity. Ability to detect change was evidenced by an approximately linear relationship between changes in FACIT-F and PtGA scores.

**Conclusions:**

FACIT-F is a reliable and valid measure for evaluating fatigue in adult patients with active AS.

*Trial registration*: ClinicalTrials.gov; NCT01786668 (registered 6 February 2013, https://clinicaltrials.gov/ct2/show/NCT01786668) and NCT03502616 (registered 11 April 2018, https://clinicaltrials.gov/ct2/show/NCT03502616).

**Supplementary Information:**

The online version contains supplementary material available at 10.1186/s41687-022-00508-0.

## Plain English summary

Ankylosing spondylitis (AS) is a disease that causes pain and stiffness in the spine. AS is a chronic disease. Most people with AS experience fatigue (a feeling of being very tired or exhausted). People with more severe AS tend to have more fatigue and a lower level of well-being. Because of this, fatigue is an important symptom to measure in studies of AS. The Functional Assessment of Chronic Illness Therapy-Fatigue (FACIT-F) is a questionnaire that measures fatigue. It has 13 questions that assess level of fatigue over a week’s time. The FACIT-F has not been studied in depth in people with AS. Therefore, we analyzed results from two clinical trials. We found that FACIT-F was a reliable and valid way to measure fatigue in adults with AS. This makes it a suitable tool to use in AS clinical trials.

**Supplementary Information:**

The online version contains supplementary material available at 10.1186/s41687-022-00508-0.

## Background

Ankylosing spondylitis (AS), sometimes referred to as radiographic axial spondyloarthritis (axSpA), is a chronic inflammatory rheumatic disease that affects the axial skeleton, potentially leading to structural and functional impairments and diminished quality of life [1–4]. Fatigue is reported by > 70% of patients with AS [5, 6] and is associated with higher levels of disease activity and functional disability, as well as worsened global wellbeing and mental health [7]. Due to its negative impact on health-related quality of life, fatigue is considered a core domain of disease assessment in AS randomized controlled trials (RCTs) [7–9].

The Functional Assessment of Chronic Illness Therapy-Fatigue (FACIT-F) scale is a questionnaire that evaluates an individual’s self-reported fatigue during their usual daily activities over the past week [10, 11]. Psychometric data analyses in patients with rheumatoid arthritis (RA) [10] and psoriatic arthritis (PsA) [12, 13] have demonstrated the reliability, validity, and internal consistency of FACIT-F. In patients with RA, FACIT-F was shown to differentiate patients according to clinical change (per American College of Rheumatology response criteria) [10]. In patients with PsA, FACIT-F was shown to differentiate patients according to disease activity (per Patient Global Assessment of Disease Activity [PtGA]) [12]. Furthermore, in patients with PsA, FACIT-F has been shown to be significantly correlated with actively inflamed joint counts and, to a lesser degree, swollen joint counts [13].

The psychometric properties of patient-reported outcomes (PROs), including FACIT-F, have been evaluated in patients with active AS treated with adalimumab or placebo [14]. As observed in patients with RA and PsA [10, 12, 13], FACIT-F had good internal consistency and was responsive to changes in clinical status (per Assessment of SpondyloArthritis International Society response criteria) [14]. However, observations were based on a relatively small sample size, and there remained scope for further psychometric analyses to increase understanding of FACIT-F as a measurement tool in AS RCTs.

Tofacitinib is an oral Janus kinase inhibitor that has been investigated for the treatment of adult patients with AS. The efficacy and safety of tofacitinib in patients with active AS, who had an inadequate response or intolerance to non-steroidal anti-inflammatory drugs (NSAIDs) have been demonstrated in phase 2 [15] and phase 3 [16] trials of 16 and 48 weeks’ duration, respectively. This post hoc analysis of data from these two clinical studies further evaluated the psychometric properties of FACIT-F in adult patients with active AS and assessed whether FACIT-F could differentiate between these patients according to disease activity.

## Methods

### Design and participants

FACIT-F validation was based on data from two RCTs of tofacitinib in patients with AS, details of which have been published previously [15, 16]. The first was a 16-week (12-week treatment, 4-week follow-up) phase 2, placebo-controlled, dose-ranging study (NCT01786668; hereby referred to as Study 1) of tofacitinib 2, 5, or 10 mg twice daily (BID) in patients (N = 207) with active AS [15]. The second was a 48-week phase 3 trial (NCT03502616; hereby referred to as Study 2) of tofacitinib 5 mg BID in patients (N = 269) with active AS. The study had a 16-week placebo-controlled double-blind phase; from Weeks 16–48, all patients received open-label tofacitinib [16].

In both studies, patients were aged ≥ 18 years, had a diagnosis of AS and fulfilled modified New York criteria for AS, documented with central reading of the radiograph of the sacroiliac joints. All patients had active disease at screening and baseline (defined as Bath AS Disease Activity [BASDAI] score ≥ 4, back pain score [BASDAI question 2] ≥ 4), and an inadequate response or intolerance to ≥ 2 NSAIDs. Patients could continue the following (stable) background therapies: NSAIDs; methotrexate (≤ 20 [Study 1] or ≤ 25 [Study 2] mg/week); sulfasalazine (≤ 3 g/day); and oral corticosteroids (< 10 [Study 1] or ≤ 10 [Study 2] mg/day of prednisone or equivalent).

This post hoc analysis used FACIT-F data from both RCTs, captured from all treatment groups (using the 13-item FACIT-F questionnaire [Additional file 1: Appendix 4, Fig. S1]) at baseline, and at Weeks 2, 4, 8, and 12 (both studies), and Week 16 (Study 2 only). The percentage of missing FACIT-F items in the studies was negligible.

### Patient and public involvement

Patients were not directly involved in the design, recruitment, or conduct of the clinical studies. Studies were conducted in accordance with the Declaration of Helsinki and International Council for Harmonisation Guidelines for Good Clinical Practice and were approved by the institutional review board and/or independent ethics committee for each study center. Written, informed consent was provided by patients.

### Psychometric analyses

#### Measurement model assessment

The FACIT-F scale is a 13-item questionnaire that evaluates an individual’s self-reported fatigue during their usual daily activities over the past week [10, 11]. The 13 items fall into either an Experience (5 items) or Impact (8 items) domain [11]. Experience items evaluate patients’ perceptions and severity of feeling, including tiredness, energy level, weakness, fatigue, and listlessness, while Impact items evaluate how fatigue impacts an individual’s daily functioning [11]. Each item is presented with a 5-point Likert scale ranging from 0 (‘not at all’) to 4 (‘very much’). After appropriate recoding so negatively phrased items are reverse scored, items are summed to calculate a FACIT-F total score ranging from 0–52, with higher scores representing less fatigue [13, 17].

The current FACIT-F measurement model is represented by both domains and total score. In the measurement model assessments, the latent construct ‘Experience’ (represented by the first-order factor f1) includes items 1, 2, 3, 4, and 7 of FACIT-F, and the latent construct ‘Impact’ (represented by the first-order factor f2) includes the remaining 8 items [11]. The latent aggregated factor (represented by the second-order factor f3) includes all Experience and Impact domains (Additional file 1: Appendix 4, Fig. S2).

Second-order confirmatory factor analysis (CFA) modeling tested the measurement structure of FACIT-F using Study 1 and 2 data (Additional file 1: Appendix 4, Fig. S2). As an indication of whether the model fits the data, the following simultaneous criteria were used [18]: 1) Bentler’s comparative fit index (CFI) > 0.90; 2) unstandardized path coefficients were statistically significant (*p* < 0.05); and 3) standardized path coefficients were > 0.40 and statistically significant (*p* < 0.05).

To support the dimensionality of the FACIT-F scale, supplemental analyses using bifactor CFA modeling were also performed. In a bifactor model, every item can be affected by only one general (overall) factor and by only one nuisance (domain) factor. Further details of the methodology are described in the Additional file 1: Appendix 1.

#### Internal consistency reliability

Internal consistency reliability was assessed using Cronbach’s coefficient alpha (α) and corrected item-to-total correlations. A Cronbach’s coefficient α ≥ 0.70 [19] and an item-to-total correlation ≥ 0.40 [18] were defined as acceptable.

#### Test–retest reliability

Intraclass Correlation Coefficients (ICCs) estimated test–retest reliability using baseline and Week 2 data and were calculated using a one-way random model (absolute agreement) [18, 20]. An ICC ≥ 0.70 was defined as acceptable [21]. Because of treatment intervention, a subgroup of ‘stable’ patients was used in the analysis, defined using PtGA scores captured during the primary studies. Patients were asked to score their overall disease activity over the last week using a numerical rating scale between 0 (‘no disease activity’) and 10 (‘very active disease’) in response to the question, *“How active was your spondylitis on average during the last week?”.* Patients were not made aware of their scores at baseline during assessment at Week 2. Test conditions and administration were consistent across visits. Two models were investigated, with only ‘stable’ patient data used in each: Model A assumed that ‘stable’ patients had the same PtGA score at baseline and Week 2; Model B was more ‘relaxed’ and assumed that ‘stable’ patients can change, but not more than 1 point in PtGA score from baseline to Week 2.

#### Convergent validity

Evidence of convergent validity (the extent to which two concepts are related to one another [22]) was evaluated by the Pearson correlation coefficients of the FACIT-F domain/total scores with the following set of PROs from Studies 1 and 2: PtGA, total back pain/nocturnal spinal pain due to AS, Short Form-36 Health Survey version 2 (SF-36v2), Bath AS Functional Index (BASFI), BASDAI, EuroQol-5 Dimension (EQ-5D) Utility Index, and AS Quality of Life (ASQoL). Although dependent on the nature of the measures being compared, and the time points evaluated (correlations are expected to be higher following a treatment intervention than at pre-treatment or baseline), under most circumstances a correlation of 0.4–0.8 may be taken as evidence of convergent validity for the target scale under consideration (in this case, FACIT-F) [18].

#### Known-groups validity

Known-groups validity was examined by evaluating differences in the reported FACIT-F domain/total scores among clinically distinct patient groups. This anchor-based approach used a repeated measures longitudinal model with the reported PtGA scores as the anchor and FACIT-F domain/total scores as the outcome. PtGA scores represented patient state from ‘no disease activity’ (PtGA score of 0) to ‘very active disease’ (PtGA score of 10).

#### Ability to detect change

Ability to detect change was based on the repeated measures longitudinal model, with change from baseline in PtGA scores at Weeks 2, 4, 8, and 12 (both studies), and Week 16 (Study 2 only), as the anchor and change from baseline in FACIT-F domain/total scores (at the same time points) as the outcome [18, 23] to examine the relationship between change from baseline in PtGA scores and change from baseline in FACIT-F domain/total scores.

#### Defining meaningful within-patient change

Meaningful within-patient change (MWPC; i.e., meaningful improvement or deterioration from the patients’ perspective) was estimated using a repeated measures longitudinal model (the same model used to define ability to detect change).

As there were not likely to be 11 distinct levels of differentiation, PtGA was transformed from a 0–10 numerical rating scale to a Patient Global Impression of Severity (PGIS) 0–4 category scale (Additional file 1: Appendix 4, Fig. S3 and Additional file 1: Appendix 3, Table S1). Thus, a 1-category difference on PGIS corresponded to a 2.5-category difference on PtGA, and a 2-category difference on PGIS corresponded to a 5.0-category difference on PtGA. As a result, MWPC was evaluated based on a 2.5-category change in PtGA, and separately, a 5.0-category change in PtGA was taken as clinically relevant change.

For FACIT-F domain/total scores, differences in mean scores between groups (numerator) divided by standard deviations at baseline (denominator) were used to estimate standardized effect sizes. These effect sizes provided a general set of thresholds or benchmarks through adjectival descriptors on the impact of an intervention, with values of 0.2 standard deviation units generally regarded as ‘small’, 0.5 as ‘medium’, and 0.8 as ‘large’ [24].

Empirical cumulative distribution functions (eCDFs) [25] were produced at the studies’ respective primary analysis time points: Week 12 (Study 1) and Week 16 (Study 2) (Additional file 1: Appendix 1, Supplemental methods).

## Results

### CFA model

Across the separate study analyses, the second-order CFA model fit the data well. CFI indices were > 0.90 at all time points (Table 1); the unstandardized path coefficients were statistically significant (*p* < 0.05), and standardized path coefficients were > 0.40 and statistically significant for all items (*p* < 0.05; Table 1). Supplemental analyses using bifactor CFA modeling supported the dimensionality of the scale (Additional file 1: Appendix 3, Table S2 and Additional file 1: Appendix 4, Figure S5).

**Table 1:** Second-order CFA model results

Time point	N	Bentler’s CFI	Standardized path coefficient, median (range)*
*Study 1*^**†**^			
Baseline	204	0.92	0.82 (0.53–0.95)
Week 2	203	0.94	0.84 (0.56–0.96)
Week 4	200	0.91	0.85 (0.57–0.96)
Week 8	198	0.95	0.85 (0.55–0.97)
Week 12	194	0.93	0.85 (0.58–0.96)
*Study 2*^**‡**^			
Baseline	268	0.92	0.81 (0.44–0.95)
Week 2	264	0.91	0.79 (0.42–0.95)
Week 4	265	0.94	0.83 (0.46–0.94)
Week 8	266	0.95	0.88 (0.49–0.95)
Week 12	264	0.93	0.86 (0.43–0.95)
Week 16	264	0.93	0.85 (0.48–0.94)

CFA, confirmatory factor analysis; CFI, comparative fit index

*All standardized path coefficients were statistically significant (*p* < 0.05)

^†^NCT01786668 (phase 2 study)

^‡^NCT03502616 (phase 3 study)

### Internal consistency reliability

In both studies, the FACIT-F domain/total scores demonstrated excellent internal consistency, with Cronbach’s coefficient α ≥ 0.88 at all time points evaluated (Additional file 1: Appendix 3, Table S3). All but one (FACIT-F Impact domain, item 8, Week 12, Study 2) of the corrected item-to-total correlations were ≥ 0.40 for all domains in both studies.

### Test–retest reliability

With both models, an acceptable (≥ 0.70) ICC was observed for the FACIT-F Experience domain (ICC: 0.75–0.86), Impact domain (ICC: 0.84–0.87), and total score (ICC: 0.86–0.89) (Additional file 1: Appendix 3, Table S4).

### Convergent validity

In both studies, all correlations were in the hypothesized directions. Correlations at baseline and Week 12 (Study 1)/Week 16 (Study 2) between FACIT-F domain/total scores and all PROs assessed were statistically significant (*p* < 0.0001) and generally ≥ 0.40, supporting convergent validity (Table 2). This trend was consistent across all other time points in both studies (Additional file 1: Appendix 3, Table S5). Across time points, the strongest correlations (0.62–0.85) were generally between FACIT-F domain/total scores and the SF-36v2 vitality domain score and ASQoL (Table 2 and Additional file 1: Appendix 3, Table S5).

**Table 2:** Correlations to assess the convergent validity of FACIT-F versus other PRO measures in patients with active AS

	Study 1*		Study 2^†^	
	Baseline	Week 12	Baseline	Week 16
*Correlations with FACIT-F Experience domain*				
PtGA	− 0.48	− 0.39	− 0.40	− 0.53
Total back pain due to AS	− 0.49	− 0.46	− 0.36	− 0.54
Nocturnal spinal pain due to AS	− 0.45	− 0.49	− 0.30	− 0.52
SF-36v2 Physical Functioning	0.59	0.57	0.53	0.60
SF-36v2 Role-Physical	0.57	0.68	0.58	0.70
SF-36v2 Bodily Pain	0.57	0.62	0.50	0.69
SF-36v2 General Health	0.51	0.59	0.51	0.63
SF-36v2 Vitality	0.77	0.85	0.75	0.85
SF-36v2 Social Functioning	0.63	0.69	0.62	0.72
SF-36v2 Role-Emotional	0.46	0.62	0.50	0.66
SF-36v2 Mental Health	0.60	0.71	0.64	0.72
SF-36v2 PCS	0.56	0.60	0.44	0.62
SF-36v2 MCS	0.61	0.74	0.64	0.76
BASFI	− 0.49	− 0.52	− 0.44	− 0.56
BASDAI	− 0.59	− 0.60	− 0.49	− 0.62
EQ-5D Utility Index	0.48	0.51	0.52	0.59
ASQoL	− 0.72	− 0.75	− 0.66	− 0.74
*Correlations with FACIT-F Impact domain*				
PtGA	− 0.46	− 0.46	− 0.39	− 0.45
Total back pain due to AS	− 0.55	− 0.55	− 0.38	− 0.50
Nocturnal spinal pain due to AS	− 0.43	− 0.57	− 0.37	− 0.51
SF-36v2 Physical Functioning	0.72	0.67	0.63	0.64
SF-36v2 Role-Physical	0.69	0.75	0.63	0.71
SF-36v2 Bodily Pain	0.62	0.64	0.54	0.68
SF-36v2 General Health	0.50	0.55	0.51	0.53
SF-36v2 Vitality	0.70	0.75	0.62	0.71
SF-36v2 Social Functioning	0.72	0.76	0.71	0.74
SF-36v2 Role-Emotional	0.60	0.73	0.56	0.66
SF-36v2 Mental Health	0.62	0.71	0.65	0.70
SF-36v2 PCS	0.64	0.64	0.50	0.61
SF-36v2 MCS	0.65	0.75	0.65	0.72
BASFI	− 0.61	− 0.62	− 0.56	− 0.60
BASDAI	− 0.59	− 0.66	− 0.52	− 0.58
EQ-5D Utility Index	0.58	0.61	0.56	0.63
ASQoL	− 0.80	− 0.82	− 0.76	− 0.78
*Correlations with FACIT-F total score*				
PtGA	− 0.49	− 0.45	− 0.41	− 0.51
Total back pain due to AS	− 0.55	− 0.54	− 0.39	− 0.54
Nocturnal spinal pain due to AS	− 0.46	− 0.56	− 0.36	− 0.54
SF-36v2 Physical Functioning	0.70	0.66	0.62	0.65
SF-36v2 Role-Physical	0.67	0.75	0.64	0.74
SF-36v2 Bodily Pain	0.63	0.66	0.55	0.72
SF-36v2 General Health	0.53	0.59	0.53	0.60
SF-36v2 Vitality	0.76	0.82	0.71	0.80
SF-36v2 Social Functioning	0.72	0.76	0.71	0.76
SF-36v2 Role-Emotional	0.57	0.71	0.57	0.69
SF-36v2 Mental Health	0.65	0.74	0.68	0.74
SF-36v2 PCS	0.64	0.65	0.51	0.65
SF-36v2 MCS	0.67	0.78	0.68	0.77
BASFI	− 0.59	− 0.60	− 0.54	− 0.61
BASDAI	− 0.62	− 0.66	− 0.53	− 0.63
EQ-5D Utility Index	0.57	0.59	0.57	0.64
ASQoL	− 0.80	− 0.82	− 0.76	− 0.80

Correlations between 0.4–0.8 were considered to be indicative of convergent validity

All correlations were statistically significant (*p* < 0.0001)

AS, ankylosing spondylitis; ASQoL, Ankylosing Spondylitis Quality of Life; BASDAI, Bath Ankylosing Spondylitis Disease Activity Index; BASFI, Bath Ankylosing Spondylitis Functional Index; EQ-5D, EuroQol-5 Dimension; FACIT-F, Functional Assessment of Chronic Illness Therapy-Fatigue; MCS, Mental Component Summary; PCS, Physical Component Summary; PRO, patient-reported outcome; PtGA, Patient Global Assessment of Disease Activity; SF-36v2, Short Form-36 Health Survey version 2

*NCT01786668 (phase 2 study)

^†^NCT03502616 (phase 3 study)

### Known-groups validity

An approximately linear relationship was observed between FACIT-F domain/total scores and PtGA scores in both studies (Additional file 1: Appendix 4, Fig. S4). In both studies, differences in the FACIT-F domain/total scores between the ‘no disease activity’ and ‘very active disease’ patient groups were statistically significant (all *p* < 0.0001; Table 3). Standardized effect sizes of all differences were large (≥ 1.17 standard deviation units), supporting known-groups validity.

**Table 3:** Known-groups validity for FACIT-F in patients with active AS

Study	Differences in the FACIT-F domain/total scores between the ‘no disease activity’* and ‘very active disease’^†^ patient groups					
	FACIT-F Experience domain score		FACIT-F Impact domain score		FACIT-F total score	
	Difference^‡^	Effect size^§^	Difference^‡^	Effect size^§^	Difference^‡^	Effect size^§^
Study 1^¶^ (N = 206)^#^	7.01	1.63	8.15	1.17	14.82	1.38
Study 2** (N = 269)^#^	6.68	1.61	7.87	1.23	14.05	1.40

AS, ankylosing spondylitis; FACIT-F, Functional Assessment in Chronic Illness Therapy-Fatigue; PtGA, Patient Global Assessment of Disease Activity

*PtGA score of 0

^†^PtGA score of 10

^‡^All differences were statistically significant (*p* < 0.0001)

^§^Effect size is the standardized effect size expressed as the difference in means between group divided by baseline standard deviation of the measure

^¶^NCT01786668 (phase 2 study)

^#^N numbers represent the total number of patients included in the model

**NCT03502616 (phase 3 study)

### Ability to detect change

**Fig. 1:**
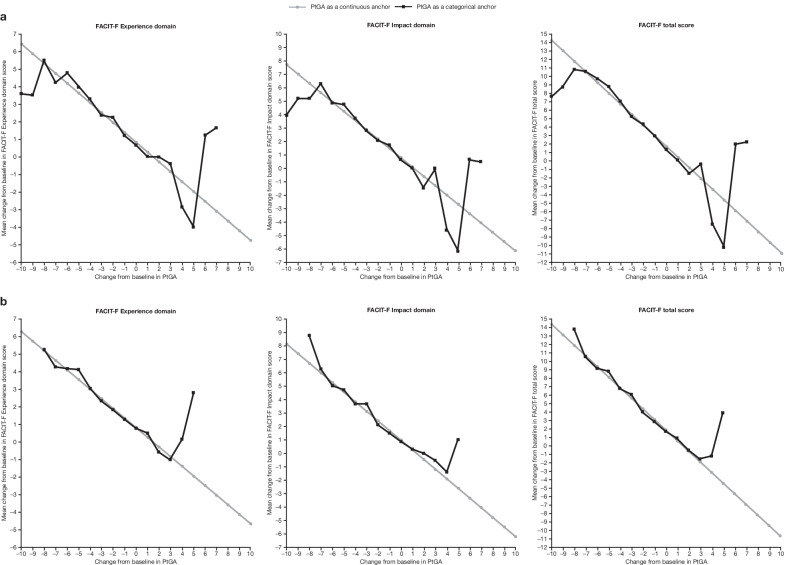
Relationship between change from baseline in FACIT-F Experience domain, Impact domain, and total scores, and change from baseline in PtGA scores in patients with active AS in **a** Study 1* and **b** Study 2^†^. *NCT01786668 (phase 2 study). ^†^NCT03502616 (phase 3 study). AS, ankylosing spondylitis; FACIT-F, Functional Assessment of Chronic Illness Therapy-Fatigue; PtGA, Patient Global Assessment

In both studies, ability to detect change was evidenced by an approximately linear relationship between changes from baseline in PtGA scores and FACIT-F domain/total scores (Fig. 1). When patients experienced a change in PtGA, values for FACIT-F domain/total scores changed accordingly. Deviations from linearity were likely due to small sample sizes at the extremes of the PtGA scores (the model with an anchor as a categorical variable does not impose any functional relationship between the outcome and anchor).

Correlations between change from baseline in PtGA and FACIT-F domain/total scores are reported in Additional file 1: Appendix 3, Table S6. Across all time points in both studies, correlations between change from baseline in PtGA scores and change from baseline in FACIT-F domain/total scores ranged from − 0.53 to − 0.31; all were statistically significant (*p* < 0.0001).

### Meaningful within-patient change

Across both studies, when a 2.5-category change in PtGA was taken as a clinically relevant change, the largest (i.e., most conservative) values estimated for MWPC for FACIT-F Experience and Impact domain scores and total score were 1.4, 1.8, and 3.1, respectively (Table 4). When a 5.0-category change in PtGA was taken as a clinically relevant change, these estimates were 2.8, 3.6, and 6.3, respectively (Table 4).

**Table 4:** MWPC estimations for FACIT-F Experience and Impact domain scores and total score

Study	Experience domain		Impact domain		Total score	
	2.5-category change in PtGA	5.0-category change in PtGA	2.5-category change in PtGA	5.0-category change in PtGA	2.5-category change in PtGA	5.0-category change in PtGA
Study 1*	1.4	2.8	1.7	3.5	3.1	6.3
Study 2^†^	1.4	2.7	1.8	3.6	3.1	6.2

FACIT-F, Functional Assessment of Chronic Illness Therapy-Fatigue; MWPC, meaningful within-patient change; PtGA, Patient Global Assessment of Disease Activity

*NCT01786668 (phase 2 study)

^†^NCT03502616 (phase 3 study)

In Study 1, standard deviations at baseline for FACIT-F Experience and Impact domain scores, and total score were 4.3, 7.0, and 10.8, respectively. Therefore, when a 2.5-category change in PtGA was used for MWPC estimation, the MWPC values corresponded to standardized effect sizes of 0.33, 0.26, and 0.29 standard deviation units, respectively, which can be interpreted as ‘small’ effects (0.26 and 0.29) or an approximately ‘small-to-medium effect’ (0.33) [24]. When a 5.0-category change in PtGA was used, MWPC values corresponded to standardized effect sizes of 0.65, 0.52, and 0.59, respectively, which can be interpreted as ‘medium’ effects.

In Study 2, standard deviations at baseline for FACIT-F Experience and Impact domain scores and total scores were almost identical to those observed in Study 1 (4.1, 6.4, and 10.0, respectively), yielding numerically similar effect sizes (data not shown).

Overall, in both studies, eCDFs for changes in FACIT-F total score showed a clear separation of curves for categories of PtGA changes with a sufficient number of available observations per category (see Additional file 1: Appendix 4, Fig. S6).

## Discussion

Fatigue is a core domain of disease assessment in AS studies [7, 8], yet data supporting the psychometric validity and reliability of the FACIT-F scale in patients with active AS are limited. This analysis of data from two clinical studies evaluated the quantitative measurement properties of FACIT-F to determine its suitability as a measure of fatigue in AS RCTs. It should be noted that the analyses reported here primarily followed US Food and Drug Administration (FDA) guidance on incorporating clinical outcome assessments (COAs) into endpoints for regulatory decision-making [26]. These guidelines are consistent with the recommendations of the OMERACT Filter 2.1, a framework designed to aid the development of core outcome measurement sets in rheumatology [27, 28].

Consistent with findings observed in a previous psychometric evaluation of FACIT-F in patients with PsA [12], the second-order and bifactor CFA modeling supported the measurement model and multidimensionality of the FACIT-F scale as an overall score with two distinguishable domains (Experience and Impact), in addition to a global domain (total score). Furthermore, in line with results seen in patients with RA [10], PsA [12, 13], and AS [14], the FACIT-F domain/total scores demonstrated excellent internal consistency across time points. The test–retest reliability analysis observed an acceptable ICC, again consistent with earlier analyses in PsA populations [12, 13].

FACIT-F domain/total scores correlated with all measured PROs, suggesting that physical and mental impacts of fatigue are closely linked to patient perception of AS. Consistent with findings in patients with AS [14] and PsA [12], the strongest correlations were seen between FACIT-F domain/total scores and SF-36v2 vitality domain scores. Interestingly, in this analysis, strong correlations were also observed between FACIT-F domain/total scores and ASQoL. Again, this is consistent with previous findings [14]. Together, these analyses suggest that patient-reported improvements in fatigue symptoms are associated with broader aspects of physical and emotional health in active AS.

Estimated MWPC values were consistent across both studies, when either a 2.5- or 5.0-category change on the anchor measure of PtGA was taken as a clinically relevant change. In line with previous findings [12], the known-groups validity analyses clearly demonstrated that the FACIT-F scale can differentiate between patients classified as having ‘no disease activity’ and ‘very active disease’ (PtGA scores of 0 and 10, respectively), as well as any pair of disease severity groups in between. FDA guidance on incorporating COAs into endpoints for regulatory decision-making defines the ability to detect change as, “*Evidence that a COA can identify differences in scores over time in individuals or groups who have changed with respect to the measurement concept”* [26]. These analyses demonstrated the sensitivity of FACIT-F to changes in PtGA scores and showed that FACIT-F is equally sensitive to increases and decreases in PtGA scores. These results were echoed in the eCDF analyses, which demonstrated that PGIS categories with more favorable change had a higher probability of having values greater than the given change in FACIT-F total score, while PGIS categories with less favorable change had a higher probability of responding less than or equal to the given change in total score. Across analyses, results from both studies were extremely similar, indicating a high degree of reproducibility.

With fatigue known to be a debilitating symptom across rheumatological diseases including AS, RA, and PsA [29], it is important to have a validated and reliable measure for evaluating fatigue across the spectrum of rheumatology patients. The consistency of findings from our evaluation in patients with AS with those previously reported in AS [14], RA [10], and PsA [12, 13] populations indicates that FACIT-F is an appropriate, uniform measure for fatigue in rheumatology.

Some limitations remain associated with these analyses. First, estimated MWPC may vary due to different methodology and natural sampling variation, and may not necessarily represent a minimal value. Second, there is no consensus as to what constitutes a meaningful change on the anchor measure. While it would have been desirable to perform test–retest reliability assessments before tofacitinib treatment began (i.e., during the screening [test] visit and baseline [retest] visit), FACIT-F assessments from the screening visit were not available. In addition, all patients had active AS; therefore, the reliability/validity of FACIT-F in patients with non-active AS remains unclear. Finally, it should be noted that the content validity and feasibility of FACIT-F in patients with AS and its validity/reliability across the spectrum of axSpA have not been empirically demonstrated to date. However, given the body of evidence available across other rheumatology indications [10, 12, 13], FACIT-F is likely to be a feasible, valid, and reliable measure of fatigue in patients with axSpA.

## Conclusions

This quantitative analysis of data from two clinical studies of tofacitinib demonstrates the validity and reliability of the FACIT-F scale as a measure of fatigue in adult patients with active AS. Overall, these findings indicate that FACIT-F is a suitable measure for use in AS clinical trials.

## Supplementary Information

**Additional file 1.** Supplemental information regarding measures, methods, and results.

## Data Availability

Upon request, and subject to review, Pfizer will provide the data that support the findings of this study. Subject to certain criteria, conditions, and exceptions, Pfizer may also provide access to the related individual de-identified participant data. See https://www.pfizer.com/science/clinical-trials/trial-data-and-results for more information.

## Abbreviations

AS: Ankylosing spondylitis
ASQoL: Ankylosing Spondylitis Quality of Life
axSpA: Axial spondyloarthritis
BASDAI: Bath Ankylosing Spondylitis Disease Activity
BASFI: Bath Ankylosing Spondylitis Functional Index
BID: Twice daily
CFA: Confirmatory factor analysis
CFI: Comparative fit index
COA: Clinical outcome assessment
EQ-5D: EuroQol-5 Dimension
FACIT-F: Functional Assessment of Chronic Illness Therapy-Fatigue
FDA: Food and Drug Administration
ICC: Intraclass Correlation Coefficient
MCS: Mental Component Summary
MWPC: Meaningful within-patient change
NSAID: Nonsteroidal anti-inflammatory drug
PCS: Physical Component Summary
PGIS: Patient Global Impression of Severity
PRO: Patient-reported outcome
PsA: Psoriatic arthritis
PtGA: Patient Global Assessment of Disease Activity
RA: Rheumatoid arthritis
RCT: Randomized controlled trial
SF-36v2: Short Form-36 Health Survey version 2
