# Supplementary material for: Functional Assessment of Chronic Illness Therapy-Fatigue is a reliable and valid measure in patients with active ankylosing spondylitis
Source: J Patient Rep Outcomes. 2022 Sep 23;6:100. doi: 10.1186/s41687-022-00508-0 (PMC9500130; doi:10.1186/s41687-022-00508-0)
Supplement: Supplementary file 1 — Additional file 1. Supplemental information regarding measures, methods, and results. [file 41687_2022_508_MOESM1_ESM.pdf]

Additional files

Appendix 1: Supplemental methods

Bifactor confirmatory analysis modeling

To investigate the dimensionality of the Functional Assessment of Chronic Illness Therapy-Fatigue (FACIT-F) scale, supplemental analyses using bifactor confirmatory factor analysis (CFA) modeling were performed, with FACIT-F represented by the global factor (latent factor fg), and Experience and Impact domains modeled as the group/nuisance factors (latent factors f1 and f2, respectively; see Appendix 4, Fig. S5). This model analyzed the relative impact of the global factor and group/nuisance factors on every item of the FACIT-F scale. If factor loadings are large on the general factor, and small on the group/nuisance factors, this would indicate that the scale is unidimensional and only a general factor should be considered (i.e., no subscales should be created). If factor loadings are large for both the general factor and the group/nuisance factors, then subscale factors should be considered (i.e., the scale should be considered as multidimensional).

Defining meaningful within-patient change: empirical cumulative distribution functions

Empirical cumulative distribution functions (eCDFs) [1] were produced at the studies' respective primary analysis time points: Week 12 (Study 1) and Week 16 (Study 2).

Patient Global Assessment of Disease Activity (PtGA) was transformed from a 0–10 numerical rating scale to a Patient Global Impression of Severity (PGIS) 0–4 category scale. By dividing PtGA values by 2.5, PtGA scores of 0 and 10 were mapped into PGIS scores of 0 and 4, respectively (see Appendix 3, Table S1). However, this created a continuous PGIS scale that

could not be used for eCDFs. Therefore, values of the continuous PGIS were rounded to the nearest integer to create a categorical PGIS scale for the eCDFs (see Appendix 3, Table S1).

When interpreting eCDFs and comparing with anchor-based modeling, it should be noted that eCDFs are based on 'completers' who had both outcomes collected (i.e., eCDFs visualize descriptive changes of an outcome in a subgroup of patients at a single time point in a study vs. an anchor-based model, which uses all available data from all patients and all time points).

Appendix 2: Supplemental results

Bifactor confirmatory analysis modeling

The comparative fit indices (CFIs) observed with the bifactor model (see Appendix 3, Table S2) were greater than those observed with the second-order CFA model (main article, Table 1). This is likely because the bifactor model has notably more parameters in the model than the CFA model (39 vs. 27 parameters, respectively).

The ratios between absolute values of a loading from the group/nuisance factor and a general factor loading are summarized in Appendix 3, Table S2. This ratio (expressed as a percentage) represents the median relative impact of the group/nuisance factor versus the impact of the general factor. The ratios observed indicated that the group/nuisance factors had a meaningful impact on the corresponding items of the FACIT-F scale (relative to the impact of the general factor).

Appendix 3: Supplemental tables

Table S1 Mapping PtGA into PGIS

**PtGA**	**PGIS (continuous)* [PtGA/2.5]**	**PGIS (categorical)†**
0	0	0
1	0.4	0
2	0.8	1
3	1.2	1
4	1.6	2
5	2	2
6	2.4	2
7	2.8	3
8	3.2	3
9	3.6	4
10	4	4

*Continuous PGIS scale used for primary MWPC analyses

†Categorical PGIS scale used for eCDFs

eCDF, empirical cumulative distribution function; MWPC, meaningful within-patient change; PGIS, Patient Global Impression of Severity; PtGA, Patient Global Assessment of Disease Activity

Table S2 Results for the bifactor CFA model using data from Study 1 and Study 2

Time point	N	Bentler's CFI	Ratio between absolute values of a loading from the group/nuisance factor and a general factor loading, %, median (range)*
**Study 1†**			
Baseline	204	0.96	31.6 (9.9–62.3)
Week 2	203	0.98	26.0 (8.8–76.6)
Week 4	200	0.94	27.1 (10.4–62.5)
Week 8	198	0.98	24.2 (3.3–59.6)
Week 12	194	0.95	31.8 (0.9–73.1)
**Study 2‡**			
Baseline	268	0.97	43.5 (21.1–73.1)
Week 2	264	0.95	30.1 (11.4–59.8)
Week 4	265	0.96	41.9 (4.5–64.1)
Week 8	266	0.97	26.9 (4.4–72.4)
Week 12	264	0.95	24.5 (7.9–158.1)
Week 16	264	0.95	43.1 (6.7–107.4)

*This ratio (expressed as a percentage) represents the median relative impact of the group/nuisance factor versus the impact of the general factor

†NCT01786668 (phase 2 study)

‡NCT03502616 (phase 3 study)

CFA, confirmatory factor analysis; CFI, comparative fit index

Table S3 Internal consistency reliability of FACIT-F in patients with active AS in Study 1 and Study 2

FACIT-F domain	Time point	N	Cronbach's coefficient α	Corrected item-to-total correlations, median (range)
**Study 1***				
Experience	Baseline	204	0.89	0.77 (0.54–0.80)
	Week 2	203	0.91	0.79 (0.59–0.86)
	Week 4	200	0.90	0.80 (0.56–0.86)
	Week 8	199	0.93	0.84 (0.65–0.87)
	Week 12	194	0.93	0.84 (0.70–0.86)
Impact	Baseline	204	0.90	0.72 (0.51–0.80)
	Week 2	204	0.90	0.73 (0.55–0.81)
	Week 4	200	0.91	0.78 (0.54–0.82)
	Week 8	198	0.91	0.75 (0.54–0.83)
	Week 12	194	0.92	0.76 (0.56–0.83)
Total score	Baseline	204	0.94	0.74 (0.53–0.83)
	Week 2	203	0.94	0.79 (0.56–0.82)
	Week 4	200	0.95	0.79 (0.56–0.86)
	Week 8	198	0.95	0.79 (0.55–0.87)
	Week 12	194	0.95	0.80 (0.58–0.87)
**Study 2†**				
Experience	Baseline	268	0.88	0.75 (0.56–0.79)
	Week 2	264	0.90	0.82 (0.51–0.85)
	Week 4	265	0.90	0.83 (0.45–0.84)

	Week 8	266	0.92	0.83 (0.59–0.86)
	Week 12	265	0.91	0.84 (0.53–0.87)
	Week 16	265	0.92	0.81 (0.63–0.86)
Impact	Baseline	269	0.88	0.71 (0.42–0.78)
	Week 2	265	0.88	0.74 (0.40–0.79)
	Week 4	265	0.89	0.74 (0.42–0.80)
	Week 8	266	0.90	0.77 (0.46–0.85)
	Week 12	264	0.90	0.75 (0.38–0.84)
	Week 16	264	0.90	0.78 (0.46–0.81)
Total score	Baseline	268	0.93	0.74 (0.43–0.80)
	Week 2	264	0.94	0.77 (0.44–0.83)
	Week 4	265	0.93	0.77 (0.43–0.83)
	Week 8	266	0.94	0.82 (0.48–0.86)
	Week 12	264	0.94	0.80 (0.46–0.87)
	Week 16	264	0.94	0.79 (0.49–0.84)

[*]NCT01786668 (phase 2 study)

[†]NCT03502616 (phase 3 study)

AS, ankylosing spondylitis; FACIT-F, Functional Assessment of Chronic Illness Therapy-Fatigue

Table S4 Test-retest reliability of FACIT-F in 'stable' patients with AS in Study 1 and Study 2

FACIT-F domain	Model A*		Model B†	
	**N (n1/n2)**	**ICC**	**N (n1/n2)**	**ICC**
**Study 1‡**				
Experience	204 (204/41)	0.75	205 (204/113)	0.79
Impact	204 (204/41)	0.84	205 (204/113)	0.87
Total score	204 (204/41)	0.86	205 (204/113)	0.89
**Study 2§**				
Experience	269 (269/85)	0.86	269 (269/179)	0.82
Impact	269 (269/85)	0.87	269 (269/179)	0.85
Total score	269 (269/85)	0.88	269 (269/179)	0.86

*Model A assumed that 'stable' patients had the same PtGA scores at baseline and Week 2

†Model B assumed that 'stable' patients had ≤ 1 point change in PtGA scores from baseline to Week 2

‡NCT01786668 (phase 2 study)

§NCT03502616 (phase 3 study)

AS, ankylosing spondylitis; FACIT-F, Functional Assessment of Chronic Illness Therapy- Fatigue; ICC, Intraclass Correlation Coefficient; N, number of patients included in the analysis; n1, number of observations at baseline; n2, number of observations at Week 2; PtGA, Patient Global Assessment of Disease Activity

Table S5 Correlations to assess the convergent validity of FACIT-F versus other PRO measures in patients with active AS in Study 1 and Study 2 at Weeks 2, 4, 8, and Week 12 (Study 2 only)

Study 1[*]	Week 2	Week 4	Week 8
**Correlations with FACIT-F Experience domain**			
PtGA	–0.45	–0.45	–0.53
Total back pain due to AS	–0.60	–0.56	–0.64
Nocturnal spinal pain due to AS	–0.57	–0.51	–0.63
BASFI	–0.62	–0.58	–0.65
BASDAI	–0.70	–0.64	–0.70
EQ-5D Utility Index	0.42	0.42	0.38
**Correlations with FACIT-F Impact domain**			
PtGA	–0.46	–0.44	–0.53
Total back pain due to AS	–0.62	–0.60	–0.64
Nocturnal spinal pain due to AS	–0.61	–0.54	–0.63
BASFI	–0.66	–0.65	–0.69
BASDAI	–0.69	–0.64	–0.69
EQ-5D Utility Index	0.53	0.50	0.48
**Correlations with FACIT-F total score**			
PtGA	–0.47	–0.46	–0.55
Total back pain due to AS	–0.63	–0.60	–0.66
Nocturnal spinal pain due to AS	–0.62	–0.55	–0.65
BASFI	–0.67	–0.64	–0.70
BASDAI	–0.72	–0.66	–0.72

EQ-5D Utility Index	0.50	0.48	0.45

Study 2†	Week 2	Week 4	Week 8	Week 12
**Correlations with FACIT-F Experience domain**				
PtGA	–0.48	–0.48	–0.52	–0.59
Total back pain due to AS	–0.47	–0.54	–0.57	–0.60
Nocturnal spinal pain due to AS	–0.45	–0.53	–0.55	–0.56
BASFI	–0.55	–0.56	–0.63	–0.61
BASDAI	–0.65	–0.66	–0.71	–0.68
EQ-5D Utility Index	-	-	-	-
**Correlations with FACIT-F Impact domain**				
PtGA	–0.46	–0.37	–0.49	–0.51
Total back pain due to AS	–0.47	–0.47	–0.55	–0.55
Nocturnal spinal pain due to AS	–0.45	–0.49	–0.55	–0.53
BASFI	–0.61	–0.59	–0.63	–0.65
BASDAI	–0.63	–0.59	–0.67	–0.63
EQ-5D Utility Index	-	-	-	-
**Correlations with FACIT-F total score**				
PtGA	–0.48	–0.44	–0.52	–0.56
Total back pain due to AS	–0.49	–0.53	–0.58	–0.60
Nocturnal spinal pain due to AS	–0.47	–0.53	–0.57	–0.56
BASFI	–0.61	–0.61	–0.66	–0.66
BASDAI	–0.66	–0.65	–0.72	–0.67
EQ-5D Utility Index	-	-	-	-

SF-36 domain and ASQoL scores were not collected at these time points

Correlations between 0.4–0.8 were considered to be indicative of convergent validity

All correlations were statistically significant ($p < 0.0001$)

*NCT01786668 (phase 2 study)

†NCT03502616 (phase 3 study)

- represent time points when the variable of interest was not collected

AS, ankylosing spondylitis; ASQoL, Ankylosing Spondylitis Quality of Life; BASDAI, Bath Ankylosing Spondylitis Disease Activity Index; BASFI, Bath Ankylosing Spondylitis Functional Index; EQ-5D, EuroQol-5 Dimension; FACIT-F, Functional Assessment of Chronic Illness Therapy-Fatigue; PRO, patient-reported outcome; PtGA, Patient Global Assessment of Disease Activity; SF-36v2, Short Form-36 Health Survey version 2

Table S6 Correlations between change from baseline in PtGA and FACIT-F Experience and Impact domain scores, and total scores in patients with AS in Study 1 and 2

**Study 1[*]**	**Week 2**	**Week 4**	**Week 8**	**Week 12**	**Week 16**
**Correlations with change from baseline in PtGA**					
Change from baseline in FACIT-F Experience domain score	–0.39	–0.35	–0.48	–0.34	-
Change from baseline in FACIT-F Impact domain score	–0.34	–0.31	–0.43	–0.38	-
Change from baseline in FACIT-F total score	–0.41	–0.37	–0.50	–0.39	-
**Study 2[†]**					
**Correlations with change from baseline in PtGA**					
Change from baseline in FACIT-F Experience domain score	–0.34	–0.36	–0.36	–0.50	–0.45
Change from baseline in FACIT-F Impact domain score	–0.31	–0.34	–0.45	–0.49	–0.42
Change from baseline in FACIT-F total score	–0.36	–0.39	–0.44	–0.53	–0.46

All correlations were statistically significant ($p < 0.0001$)

[*]NCT01786668 (phase 2 study)

[†]NCT03502616 (phase 3 study)

- represent time points when the variable of interest was not collected

AS, ankylosing spondylitis; FACIT-F, Functional Assessment of Chronic Illness Therapy-Fatigue; PtGA, Patient Global Assessment of Disease Activity

Appendix 4: Supplemental figures

Fig. S1 The FACIT-F scale.

FACIT Fatigue Scale (Version 4)

Below is a list of statements that other people with your illness have said are important.
Please circle or mark one number per line to indicate your response as it applies to the past 7 days.

Item ID		Not at all	A little bit	Some-what	Quite a bit	Very much
H17	I feel fatigued	0	1	2	3	4
H12	I feel weak all over	0	1	2	3	4
An1	I feel listless ("washed out")	0	1	2	3	4
An2	I feel tired	0	1	2	3	4
An3	I have trouble starting things because I am tired	0	1	2	3	4
An4	I have trouble finishing things because I am tired	0	1	2	3	4
An5	I have energy	0	1	2	3	4
An7	I am able to do my usual activities	0	1	2	3	4
An8	I need to sleep during the day	0	1	2	3	4
An12	I am too tired to eat	0	1	2	3	4
An14	I need help doing my usual activities	0	1	2	3	4
An15	I am frustrated by being too tired to do the things I want to do	0	1	2	3	4
An16	I have to limit my social activity because I am tired	0	1	2	3	4

The FACIT-F scale is owned and copyrighted by, and the intellectual property of, David Cella, PhD. Reproduced with permission from David Cella, PhD. All items are summed, after appropriate recoding so negatively phrased items are reverse scored, to calculate a FACIT-F total score ranging from 0–52, with higher scores representing less fatigue. Abbreviation: FACIT-F, Functional Assessment of Chronic Illness Therapy-Fatigue

Fig. S2 FACIT-F second-order CFA model.

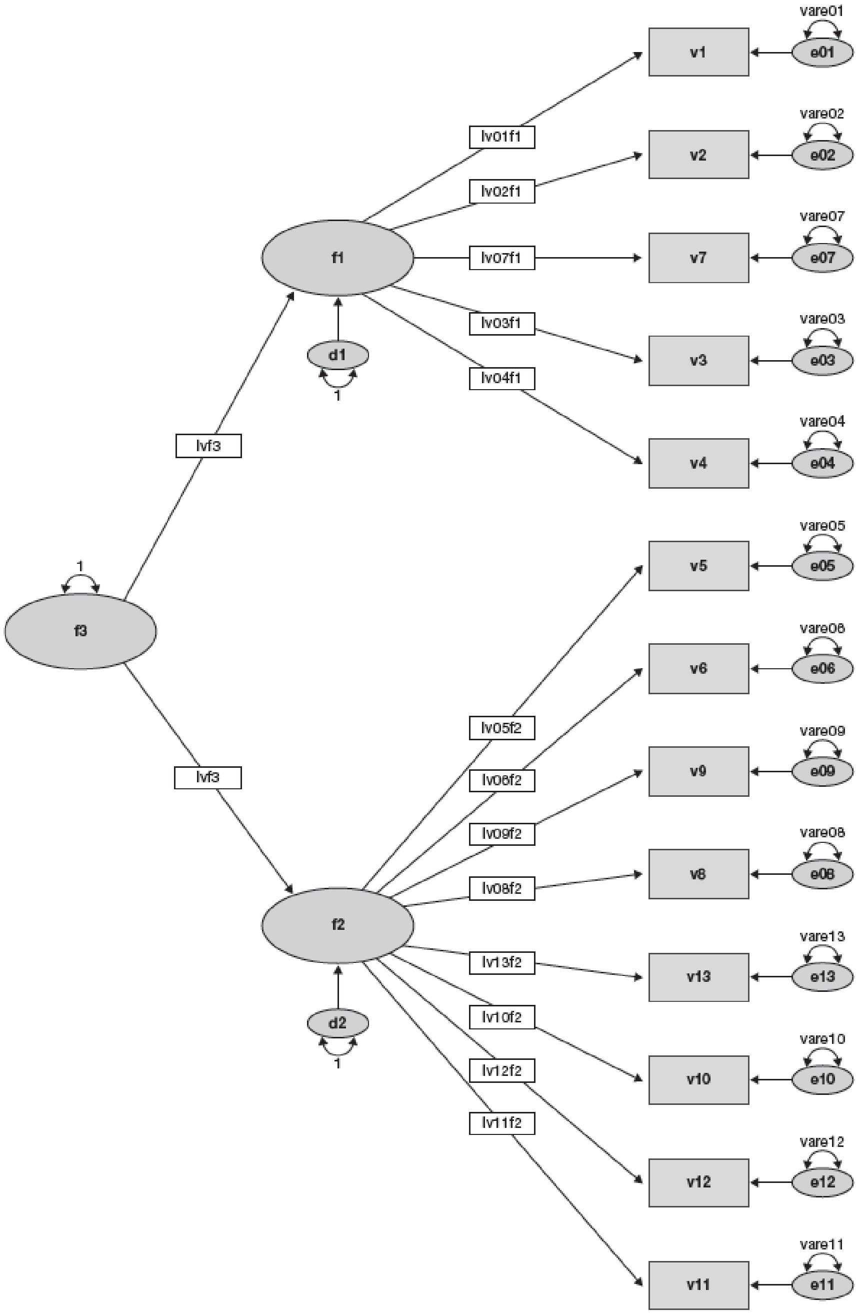

In the CFA model, the Experience and Impact domains were represented by latent (unobserved) variables f1 and f2, respectively. The second-order aggregate latent factor f3 affects (subsumes) both factors f1 and f2. v1–13 represent the items of the FACIT-F scale. The factor loadings were represented by 'lvf' and 'lf' path coefficients (e.g., lvf3f2 represents the path coefficient [loading] from factor f3 to f2, and lv02f1

represents the path coefficient [loading] from factor f1 to item or variable 2). The disturbance terms for the factors were represented by ‘d’ (e.g., d2 represents the disturbance terms associated with factor 2). The error terms for the observed items were represented by ‘e’ (e.g., e1 represents the error term associated with item or variable 1). Abbreviations: CFA, confirmatory factor analysis;
FACIT-F, Functional Assessment of Chronic Illness Therapy-Fatigue

Fig. S3 Example of PGIS scales.

Please choose the response below that
best describes the severity of your
<SYMPTOM/OVERALL STATUS/ETC.>
over the past week.

☐ None
☐ Mild
☐ Moderate
☐ Severe
☐ Very severe

PGIS scored from 0 (none) to 4 (very severe). Abbreviation: PGIS, Patient Global Impression of Severity

Fig. S4 Relationship between reported FACIT-F Experience domain, Impact domain, and total scores, and reported PtGA scores in patients with active AS in **a** Study 1* and **b** Study 2†.

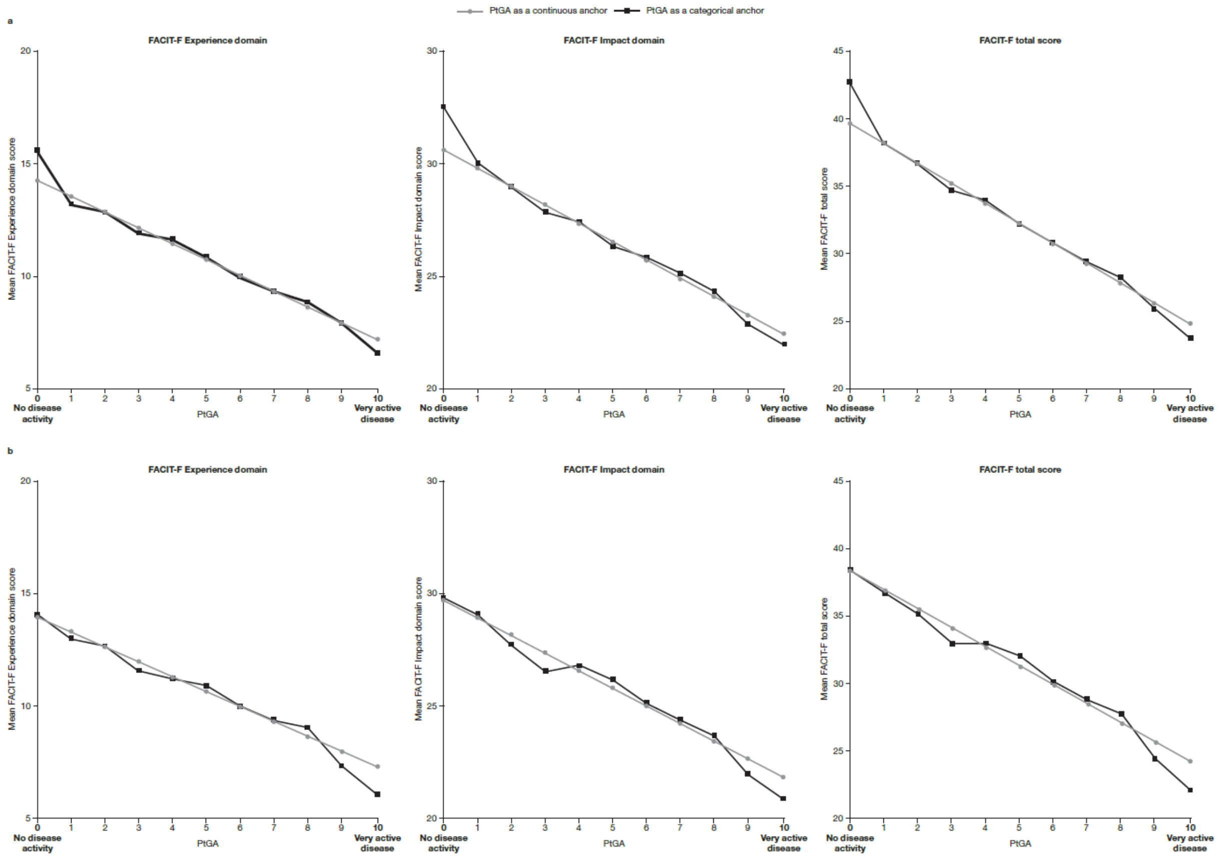

*NCT01786668 (phase 2 study). †NCT03502616 (phase 3 study). Abbreviations: AS, ankylosing spondylitis; FACIT-F, Functional Assessment of Chronic Illness Therapy-Fatigue; PtGA, Patient Global Assessment of Disease Activity

Fig. S5 Bifactor CFA model.

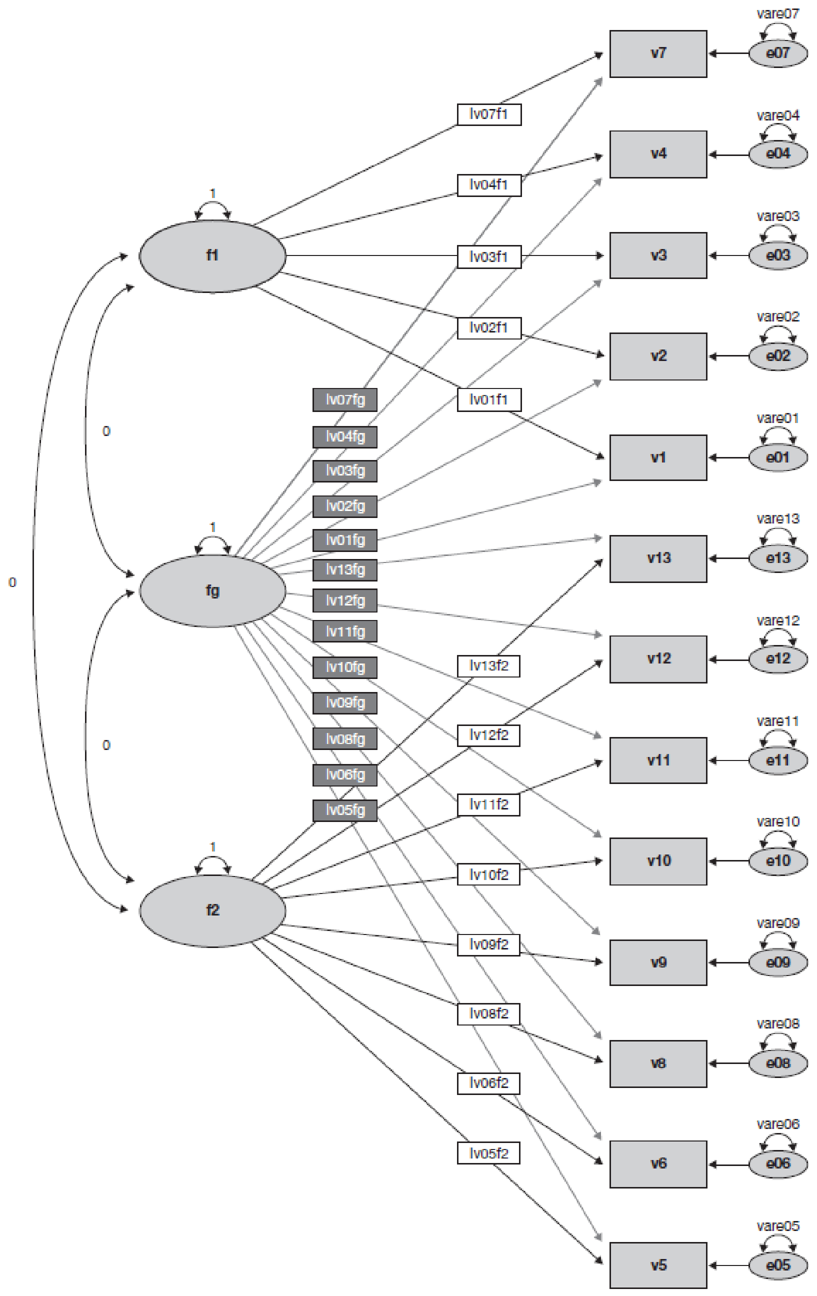

The latent factor fg represents global factor for FACIT-F scale and f1 and f2 represent group/nuisance factors (which should be interpreted as residuals relative to the general factor fg); v1–13 represent the items of the FACIT-F scale. Abbreviations: CFA, confirmatory factor analysis; FACIT-F, Functional Assessment of Chronic Illness Therapy-Fatigue

Fig. S6 eCDF of change in total FACIT-F score in **a** Study 1* at Week 12 and **b** Study 2† at Week 16.

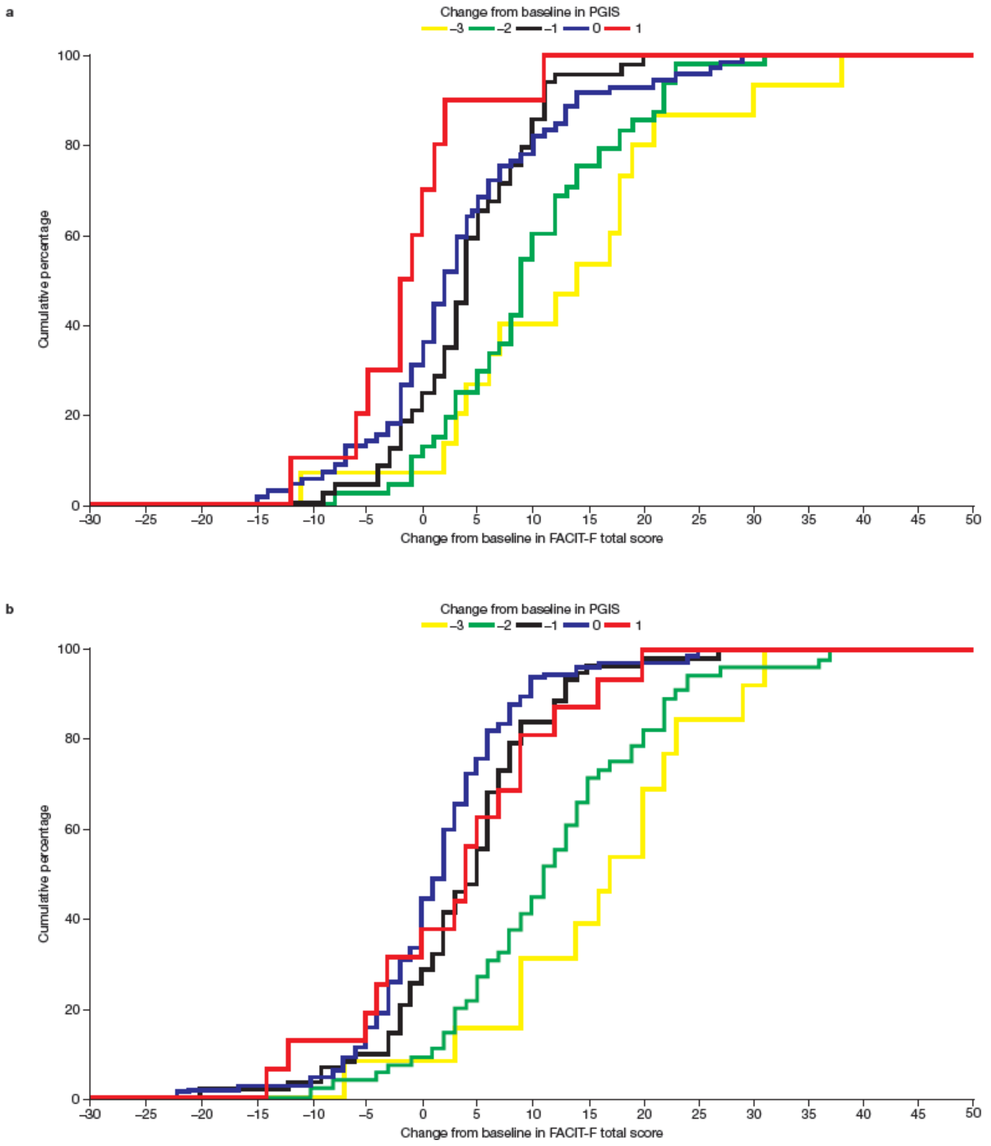

*NCT01786668 (phase 2 study). †NCT03502616 (phase 3 study). Abbreviations: eCDF, empirical cumulative distribution function; FACIT-F, Functional Assessment of Chronic Illness Therapy-Fatigue; PGIS, Patient Global Impression of Severity

Reference

1. Gibbons, J.D. and S. Chakrabarti, *Nonparametric statistical inference*. 5th ed. 2011, Boca Raton, Florida: Chapman & Hall/CRC Press.
